# What do we actually know about the biomechanics of pregnancy and labour? A systematic scoping review

**DOI:** 10.1371/journal.pone.0337595

**Published:** 2025-12-01

**Authors:** Anastasia Topalidou, Lauren Haworth, Raeesa Jassat, Morgan Hawcroft-Hurst

**Affiliations:** 1 School of Nursing and Midwifery, University of Lancashire, Preston, United Kingdom; 2 School of Health, Social Work and Sport, University of Lancashire, Preston, United Kingdom; 3 School of Health, Social Work and Sport, University of Lancashire, Westlakes, United Kingdom; 4 School of Biomedical, Nutritional, and Sport Sciences, Faculty of Medical Sciences, Newcastle University, Newcastle Upon Tyne, United Kingdom; Iran University of Medical Sciences, IRAN, ISLAMIC REPUBLIC OF

## Abstract

Pregnancy and childbirth involve profound biomechanical transformations, adaptations, and functional demands on the maternal body. Although biomechanical complications have been identified as a major contributor to maternal morbidity and mortality, this remains one of the most under-researched areas in perinatal health. This systematic scoping review aimed to map and synthesise existing literature on the biomechanics of pregnancy and labour. Following Arksey and O’Malley’s framework and PRISMA-ScR guidance, comprehensive searches of MEDLINE, EMBASE, and MIDIRS were conducted up to May 2025. Eligible sources were peer-reviewed empirical studies assessing musculoskeletal, kinematic, kinetic, postural, or dynamic parameters in pregnant or labouring women. Titles, abstracts, and full texts were screened against predefined eligibility criteria. Data were charted using a structured extraction form and synthesised narratively across key biomechanical themes. Eighty-seven studies were included, all of which focused on pregnancy. No studies conducted during labour were identified. Most were observational with small sample sizes and limited diversity. Ethnicity was reported in only one study. Four key themes emerged: (1) Posture and spinal curvature, (2) Gait and locomotor analysis, (3) Functional tasks and interventions, and (4) Balance and stability. Findings showed high individual variability and no consistent biomechanical pattern across pregnancy. Real-world, neuromuscular, and labour-related biomechanics remain largely unexplored. This review underscores a critical gap in perinatal research: while biomechanical adaptations during pregnancy have been increasingly studied, labour remains entirely unexamined from a biomechanical perspective. Current evidence is fragmented, methodologically narrow, and lacks diversity, offering limited clinical relevance. We are effectively operating in a biomechanical vacuum, without empirical data to guide safer, more efficient, and personalised birth practices. Existing clinical approaches rely heavily on tradition, anecdotal experience, and untested theoretical assumptions. Addressing this evidence void, particularly in labour biomechanics and ethnic representation, is essential to improve perinatal outcomes and support equity in maternal care.

## Introduction

Pregnancy and childbirth are defining periods in a person’s life, during which the body undergoes a series of orchestrated adaptations affecting nearly every physiological system [1–3]. These include significant physiological and biomechanical changes aimed at accommodating the growing foetus and preparing the body for delivery [4,5]. From a biomechanical perspective, pregnancy involves adaptations such as increased ligament laxity, spinal realignment, changes in body mass distribution, and shifts in the centre of gravity (CoG) [6–9]. These changes necessitate adjustments in posture and mobility [6,7], which are critical for maintaining balance, reducing physical strain [10–12] and supporting the well-being of both the mother and the foetus.

As the pregnancy progresses to labour, the focus shifts to the biomechanics of childbirth, involving the dynamic interaction between the woman’s body and the foetus. This process encompasses the engagement of the foetus with the maternal pelvis [13,14], and changes in pelvic orientation and joint mobility [9,15] that facilitate descent through the birth canal [13,14]. During the second stage of labour, intra-abdominal pressure is generated through maternal voluntary effort, co-ordinated with involuntary uterine contractions [13,16,17]. The mechanical forces involved in labour and birth are multifaceted, combining musculoskeletal adaptations, adjustments in neuromuscular control, and physiological responses to the advancing stages of childbirth [16–18].

The biomechanics of pregnancy typically refers to the study of the structural, mechanical, and functional changes of the maternal body in response to gestation. It examines how the musculoskeletal system adapts to the demands of carrying a growing foetus, including alterations in posture, balance, and mobility. This field also explores the forces exerted by and on the body, and how these interact with physiological changes, such as ligament laxity, increased maternal mass, and shifts in the CoG [4,6,7,9]. Conversely, the biomechanics of labour and birth focuses on the study of the mechanical and functional processes involved as the body prepares for and facilitates the delivery of a baby. It encompasses the analysis of maternal positioning and movements, as well as pelvic dynamics that support foetal alignment. Additionally, in the second stage, it examines the mechanics of uterine contractions and maternal pushing, alongside the effect of birthing positions on foetal descent, mechanical efficiency, maternal comfort, and outcomes for both the woman and the baby [13,15,18–21]. The biomechanical efficiency of labour and birth also involves the energy expenditure and mechanical stress on maternal tissues, which are critical for developing knowledge and interventions to reduce the risk of injury and complications [17,18]. Moreover, biomechanics investigates how an individual’s motor control, anatomical and musculoskeletal characteristics, such as pelvic geometry, joint mobility, alignments or angulations across the body, influence labour mechanics. It aims to provide a deeper understanding of the biomechanical challenges and solutions during this critical life event, and to determine optimal practices based on personal characteristics and the interactions between mother and foetus, as well as the internal and external forces involved [18,22,23].

The implications of biomechanical factors extend beyond adaptation; they are directly linked to outcomes. Maternal and neonatal mortality rates remain unacceptably high worldwide [15,24,25], with figures rising again in both low- and middle-income countries (LMICs) and high-income countries (HICs) — an issue described as a *“major scandal”* and a *“global failure”* [26]. In the United Kingdom (UK) for instance, maternal mortality rose to its highest level in 20 years [27]. Sustainable Development Goal (SDG) 3.1, which targets the reduction of maternal mortality, stands as the only SDG that has notably failed [26,28], with biomechanical complications being a major contributing factor [15,20]. Despite this critical impact, biomechanics remains one of the most under-researched areas in perinatal health. To date, no attempt has been made to systematically synthesise biomechanical research in pregnancy and labour. Bringing together the available evidence is essential to establish what is currently known, to identify gaps, and to lay the foundation for future investigation that can better inform maternal health outcomes.

The purpose of this systematic scoping review is to comprehensively identify and map the existing literature on the biomechanics of pregnancy and labour, including adaptations and assessment methods. It aims to identify and synthesise studies that explore musculoskeletal, kinematic, kinetic, postural, and dynamic aspects of biomechanics, exclusively in pregnant and labouring women.

## Methods

This systematic scoping review aimed to map out the key concepts and evidence available on the biomechanics of pregnancy and labour. It specifically focused on studies that investigated comprehensive biomechanical analyses during these periods, including musculoskeletal, kinematic (movement), kinetic (forces), postural, and dynamic assessments, exclusively involving pregnant and labouring women.

The protocol for this review was structured using the methodological framework for scoping reviews as described by Arksey and O’Malley [29] and followed the guidance for conducting systematic scoping reviews as outlined by Peters et al. [30]. Systematic scoping reviews are designed to identify and map the primary concepts underpinning a research area, summarise the main sources of evidence, and outline the types of evidence available [29–31]. Unlike systematic reviews, which are narrower in scope, focus on specific questions and often assess the quality of included studies, scoping reviews allow for a broader enquiry and the methodology can be more flexible, making them suitable for emerging fields or complex subjects where many studies might not focus strictly on intervention effectiveness [30,32,33].

To promote transparency, reduce bias, and support open science practices, the protocol was prospectively registered on the Open Science Framework (OSF) [Registration DOI https://doi.org/10.17605/OSF.IO/Z7KJW ] and is publicly available at: https://osf.io/z7kjw [34].

Based on the aim of this review, the review question was: “What biomechanical changes, adaptations, and responses occur during pregnancy and labour, and how are these assessed and quantified in terms of musculoskeletal, kinematic, kinetic, postural, and dynamic parameters in pregnant and labouring women?”. The secondary questions included, but were not limited to: “How do different stages of pregnancy and associated changes affect the biomechanical profiles of pregnant women?”, “How do various stages of labour, labour positions, and practices influence the biomechanical profiles of labouring women and the outcomes of labour?”, “What methodologies and technologies are primarily used to assess and investigate biomechanics during pregnancy and labour?”, and “Where are the significant gaps in the current research on biomechanics during pregnancy and labour?”.

### Search strategy

A preliminary search of two databases [(Ovid MEDLINE(R) and Epub Ahead of Print, In-Process & Other Non-Indexed Citations and Daily) and (Ovid Embase)] was conducted to identify adequate search terms related to the scope of this review. Following this, an intensive literature search was conducted in the following electronic databases (from the earliest date in each database):

a) OVID Embase (Ovid) <1974 to May 01, 2025> (Date of search: 02/05/2025)
b) Ovid MEDLINE(R) ALL <1946 to May 01, 2025> (Date of search: 02/05/2025)
c) Maternity & Infant Care Database (MIDIRS) (Ovid) <1971 to April 29, 2025> (Date of search: 02/05/2025)

In order to extract all available data, the search strategy was kept necessarily broad combining the terms: “biomechanic*” OR “*motion capture*” OR “*motion analysis*” OR “*movement analysis*” OR “*body tracking*” AND “*childbirth*” OR “*birth*” OR “*labo?r*” OR “pregnan*”. The search strategy was limited to English language only. The full search strategy, including syntax, and the number of hits for each query in each database are detailed in the supporting information (S1 Appendix).

### Inclusion/exclusion criteria

Records were not excluded on the grounds of quality, geographical location, or date of study, as the purpose was to scan the literature and determine the up to date reported studies, knowledge, and gaps.

#### Inclusion criteria.

a) Study population: Only studies involving human participants who were pregnant during their antenatal period or during labour.
b) Study focus: Studies that investigated musculoskeletal biomechanics during pregnancy and/or childbirth, which included musculoskeletal, kinematic, kinetic, postural assessments, and adjustments, as well as dynamic responses.
c) Type of studies: Original research studies that provided primary data with clear methodology and reporting of results, and had undergone peer-review.

#### Exclusion criteria.

a) Non-relevant population: Studies reporting solely on non-pregnant individuals or non-labouring populations.
b) Non-relevant topics: Studies that focused on cellular, tissue-level, or organ-specific research without direct relevance to musculoskeletal biomechanics (e.g., studies solely focused on the cervix, placenta, or cellular mechanisms). Studies reporting anatomical measurements (e.g., stance width, girth, or base of support) were included only if these were linked to functional biomechanical assessments such as balance, postural control, or motion analysis.
c) Non-relevant types of studies: Records that did not report primary data, had not undergone peer-review, or lacked clear methodology and reporting of results, including reviews, protocols, methodological evaluations (e.g., inter- and intra-rater reliability), editorials, opinion papers, letters, commentaries, book chapters, and preprints.
d) Computational and modelling studies: Studies focused exclusively on computational modelling, finite element analysis, digital human modelling, and avatars that did not include direct biomechanical measurements or assessments on human subjects.
e) Language and accessibility: Records with no full text available in English. Conference abstracts without accessible full texts, and publications missing abstracts (at the first screening stage), or full texts (at the second screening stage).

### Screening process

In total, two stages of deduplication were performed. Initially, all exported citations were uploaded into EndNote (version 21, Clarivate Analytics, Philadelphia, PA), where duplicate articles were identified and removed (1^st^ stage of deduplication). Then the final EndNote dataset was exported and uploaded into Rayyan® (https://www.rayyan.ai/), a web-based software platform for systematic reviews [35], where further duplicates were detected and removed (2^nd^ stage of deduplication).

The review process consisted of two levels of screening. At the first stage, using Rayyan®, the titles and abstracts of identified articles were screened according to the inclusion and exclusion criteria by two sets of researchers (RJ, AT; AT, LH) [35]. Disagreement between reviewers during this stage resulted in the article’s inclusion for full text review. All attempts were made to obtain the full texts of selected articles. Records that were not available in full text, or those that the authors were unable to obtain, are listed in supporting information (S2 Appendix). In the second stage, full text screening was conducted by three sets of reviewers (MHH, AT; MHH, LH; AT, LH) to identify eligible studies. A third reviewer helped resolve any disagreements as necessary. Articles that did not meet the inclusion criteria at this stage were documented, alongside the specific reasons for their exclusion.

The selection process followed the recommendations in the Preferred Reporting Items for Systematic Reviews and Meta-Analyses Extension for Scoping Reviews (PRISMA-ScR) checklist [36] (see supporting information, S3 Appendix) and was mapped using the PRISMA flow diagram [30,37].

### Charting data and analysis

Data were extracted from the selected studies using a data extraction form created by the authors. The following information was extracted during the charting process:

a) Study information: Author(s), publication year, country, study aim/objective, type of study
b) Participants/characteristics of the study population: Sample size, age, weight, height, ethnicity, weeks of gestation and other demographics
c) Method(s) used for the biomechanical assessment/analysis/investigation.
d) Type of assessment [as described by the author(s)]
e) Description of assessment/analysis/investigation
f) Type of data/variables analysed/presented in the record
g) Key findings
h) Limitations (if any) as described by the author(s)
  i) Comments

The data extraction form was initially pilot tested with a small subset of included studies to ensure its suitability. Based on this pilot, necessary adjustments were made to accommodate any data that did not fit into the existing fields, by creating new fields that more effectively addressed the scope of this review. Following the pilot, the research team convened to assess the consistency of the data extraction approach with the research question and objectives. A consensus was then reached regarding the final fields/categories to be used for data extraction. After the successful pilot test, the remaining included studies were systematically reviewed, and data were extracted and mapped into the established fields by three sets of reviewers (MHH, AT; MHH, LH; AT, LH).

As this study is a scoping review, the data analysis included a narrative synthesis of the extracted data. Findings from the included studies were organised and presented in tabular form, offering an overview of the key themes and patterns across the biomechanical aspects of pregnancy and labour.

### Ethical considerations

As this study involved secondary research, no ethical approval was required. All data used were obtained from previously published studies, for which the original authors had secured the necessary ethical approvals.

## Results

**Fig 1:**
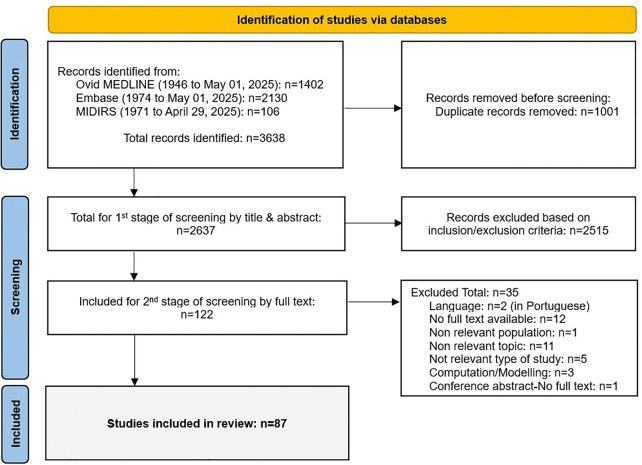
PRISMA flow chart illustrating the literature search and screening process.

A total of 3638 records were identified through the database searches (EMBASE, MEDLINE and MIDIRS). After the removal of duplicates, 2637 records were screened by abstract and title, and 122 were taken forward for full text review. After the final full text selection, 87 records remained and were included in this review (Fig 1).

### Study characteristics

The characteristics of the 87 included studies are summarised in S1 Table. The majority were observational in design (n = 82), with only five studies employing other methodologies: four experimental studies and one retrospective analysis of a randomised controlled trial. Among the observational studies, longitudinal follow-up designs were the most prevalent (n = 36), followed by longitudinal follow-up studies with a non-pregnant control group (n = 23), and cross-sectional comparative studies (n = 16). The remaining 12 studies encompassed a range of other designs, as detailed in S1 Table. The full dataset is also provided as supporting information (S1 Table) for ease of access.

**Fig 2:**
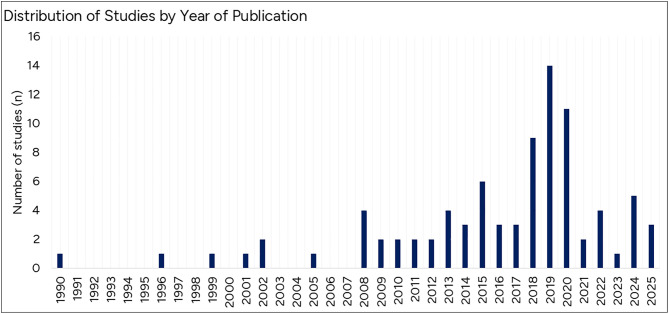
Distribution of studies on the biomechanics of pregnancy by year of publication.

Although the first study assessing biomechanics during pregnancy was in 1990 [38], there were numerous publication gaps between 1990 and 2007. From 2008 to 2017, there was a relatively stable but modest level of research activity. A clear peak in publication frequency occurred between 2018 and 2020. While studies have continued to be published since then, the annual output has declined, and has not returned to peak levels (Fig 2).

The included studies were undertaken in the following countries: the United States of America (USA) (n = 24), Poland (n = 9), Japan (n = 6), Australia (n = 5), Portugal (n = 5), Brazil (n = 4), Turkey (n = 4), France (n = 4), Canada (n = 3), the Netherlands (n = 3), China (n = 3), Spain (n = 2), Norway (n = 2), Taiwan (n = 2), Benin (n = 2), South Africa (n = 2), Czech Republic (n = 2), Germany (n = 2), Switzerland (n = 1), India (n = 1) and Pakistan (n = 1).

The studies included in the review generally featured small sample sizes, ranging from as few as two participants [39], to a maximum of 131 participants [40]. Fifty percent of the studies recruited 23 or fewer participants; specifically, 14% (n = 12) involved 10 or fewer participants, 36% (n = 31) included 11–20 participants, 28% (n = 24) recruited 21–30 participants, and 10% (n = 9) involved 31–40 participants. Although 87 studies were included in this review, analysis of participant characteristics and sample sizes suggests that several publications likely drew from overlapping cohorts [41–58]. Across all studies, a total of 2,277 participants were reported. However, after accounting for likely duplicate reporting, an estimated 2,033 unique pregnant participants were included.

Although the majority of included studies reported participant age, height, weight, or body mass index (BMI), ethnicity was almost entirely unreported. Of the 87 studies, only one explicitly reported participant ethnicity, stating that all participants were Caucasian [59]. Another study described participants as Japanese [60], although this reflects nationality rather than ethnicity. Additionally, two studies implied participants’ ethnic backgrounds through geographical location, such as West African populations [61,62] but none provided detailed ethnic breakdowns or further demographic specifics.

Kinematic analyses emerged as the most frequently employed method (n = 47), closely followed by kinetic analyses (n = 42). Postural evaluations were conducted in 18 instances, while surface electromyographic (sEMG) assessments appeared in eight studies. Balance assessments were reported in six studies, and spatiotemporal gait parameters were evaluated in four. Biomechanical asymmetry was examined in only one study.

Notably, all 87 included studies were conducted during pregnancy, involving pregnant women. No studies were identified that investigated biomechanical parameters during labour, including labouring women.

### Biomechanics of pregnancy

#### Narrative synthesis.

The reviewed literature presents a diverse range of biomechanical adaptations during pregnancy, in response to the anatomical, physiological, and functional demands of gestation. Findings are heterogeneous but cluster around four key domains (Table 1): posture and spinal curvature, gait and locomotor analysis, functional tasks including interventions, and balance and stability.

**Table 1:** Summary of the themes and corresponding studies included in this review.

Theme	Studies
**Posture and spinal curvature**	
Standing	[38,42,48,53,58,64–67,69]
Sitting	[48,70]
Birth position	[39,45,46,71,72]
**Gait and locomotor analysis**	
Spatiotemporal gait parameters	[1,10,43,50–52,55,56,73–87]
Pelvic and thoracic kinematics	[1,43,52,55,75,80,86,88,89]
Plantar pressure and foot loading	[40,77,81,90–97]
Fall risk and neuromechanical adaptations	[52,56,78,82,98–100]
Neuromuscular control and joint-level adaptations	[44,84,98,101,102]
**Functional tasks and interventions**	
Functional tasks: Sit-stand/ Stand-sit	[49,57,58,60,103–107]
Stair negotiations	[53,54,108]
Walking initiation and transitional movements	[62,78,105]
Load carrying tasks	[47,61,62]
Task-specific balance responses	[47,61,62,109–112]
Interventions	[113–115]
ROM-specific tasks	[42,47,109]
**Balance and stability**	
	[9,41,55,59,77,82,116–120]

#### Posture and spinal curvature.

Postural adaptations during pregnancy are highly individualised and biomechanically complex, influenced by factors such as gestational stage, body morphology, and task demands. While the literature consistently highlights changes in spinal curvature and pelvic orientation, findings remain heterogeneous, with no single trajectory or “optimal” postural response universally observed across individuals or trimesters.

**Standing:** The biomechanical adaptations of standing posture during pregnancy are complex and heterogeneous, with evidence revealing both shared trends and considerable inter-individual variability. Across the reviewed studies, changes in spinal alignment, including lumbar and cervical lordosis, thoracic kyphosis, trunk flexion angle, postural alignment, sacral inclination, and pelvic orientation, are inconsistently reported. This reflects the influence of methodological differences, gestational timing, body mass, and individuals’ characteristics, among other factors.

Specifically, lumbar lordosis during standing does not follow a uniform trajectory across gestation and appears to depend on individual characteristics. In a longitudinal study, Moore et al. [38] found that 56% of pregnant participants exhibited a decrease in lumbar curvature from the first to the second trimester, while 44% showed increased lordosis by the third trimester. Pauk and Swinarska [63] documented a statistically significant increase in both thoracic kyphosis and lumbar lordosis between the second and third trimester, with kyphosis rising from 53.2° to 57.3° and lordosis from 40.0° to 44.2°. These increases were positively correlated with BMI, suggesting that maternal body mass plays a role in amplifying spinal curvature. However, Schroder et al. [64], reported a decrease in lumbar lordosis from 51.1° to 49.4° over the same period, and no consistent trend in sacral inclination. Bivia-Roig et al. [41] further supported this inconsistency by reporting no significant differences in lumbar curvature between pregnant, postpartum, and nulliparous participants in standing, despite observing increased trunk extensor muscle activity in pregnant women, possibly reflecting compensatory neuromuscular demands. Gilleard et al. [48] also observed no consistent sagittal plane changes in standing trunk posture across pregnancy, suggesting that some individuals adopt flatter spinal curves while others show increased extension.

Pelvic orientation also yielded inconsistent findings. Opala-Berdzik et al. [65] found no significant change in sacral inclination from early to late pregnancy, whereas Catena and Wolcott [66] documented an increase in lumbopelvic angle up to the second trimester, which then plateaued. This adaptation appeared to reduce the standing hip moment and support the maintenance of centre of mass (CoM) alignment over the hips. Catena et al. [67] also reported anterior and superior shifts in both trunk and whole-body CoM across gestation, with lateral displacements varying in direction between individuals. These findings reinforce the view that postural adaptation is highly individualised and strategically modulated to maintain stability.

Task-specific studies discussed in detail below add further context. Paul et al. [68] quantified a 2.8-fold increase in hip joint moments during standing work postures between 10 and 40 weeks of gestation, with approximately half of this load increase attributable to postural adjustments. Gilleard et al. [48] reported reduced thoracic and thoracolumbar mobility during standing flexion and rotation tasks as pregnancy progressed, suggesting increased rigidity or neuromuscular restraint in the upper trunk. Clinical and metabolic conditions may further influence postural responses. In a comparative study, Valerio et al. [69] found that pregnant women with type 1 diabetes exhibited increased cervical lordosis, thoracic kyphosis, and lumbar lordosis compared to healthy controls. Interestingly, this group also demonstrated reduced pelvic anteversion and forward head posture, suggesting a distinct postural profile possibly shaped by altered muscle tone or metabolic influences.

These findings demonstrate that standing posture during pregnancy is not governed by a singular biomechanical pattern. Instead, it reflects a dynamic equilibrium between structural adaptation, body morphology, and compensatory strategies.

**Sitting:** Evidence on sitting posture adaptations during pregnancy is limited, with only two studies directly addressing this area. Gilleard et al. [48], using a static sitting protocol, reported no statistically significant changes in sagittal-plane upper body alignment when sitting, although there was a non-significant trend toward flatter spinal curves as pregnancy progressed. In contrast, Lee at al. [70], studying dynamic workstation tasks, identified clear postural differences between pregnant and non-pregnant women across various seated workstation configurations. Pregnant participants exhibited greater trunk flexion, increased thigh angles, and more forward-reaching upper arms, likely reflecting compensatory adjustments for abdominal enlargement. These changes increased biomechanical loading on the lower back and were influenced by workstation design. Specifically, a forward-sloping seat (10° inclination) reduced trunk flexion and was subjectively preferred, whereas a flat seat (0°) was associated with greater discomfort. These findings suggest considering ergonomic factors in relation to pregnancy-related morphological changes, particularly when aiming to optimise seated posture and reduce musculoskeletal strain during sedentary work.

**Birth position (experimental setting):** Evidence on the biomechanics of birthing positions during pregnancy is limited to five experimental studies conducted with small samples of pregnant women (ranging from 13 to 23 participants), all in late gestation (>32 weeks) and not in labour [39,45,46,71,72]. Only a limited number of birthing positions were assessed, primarily variations of squatting, supine lithotomy, and the McRoberts’ manoeuvre, with most analyses focusing on sagittal-plane spinal curvature, hip angles, and pelvic alignment. These studies used motion capture and spinal curvature measurement systems to analyse static postures assumed in controlled environments; they were not conducted during labour, and they did not account for the dynamic physiological and mechanical influences of labour, uterine contractions, or foetal interaction.

Across studies, squatting postures demonstrated substantial interindividual variability in lumbar curvature and pelvic tilt. Tiptoe squatting induced greater lumbar lordosis and less favourable pelvic alignment compared to flat-foot squatting, which was more frequently associated with kyphosis or flattened spines [45]. However, nearly all participants (11 out of 13) spontaneously adopted the tiptoe position during initial testing, despite it being biomechanically suboptimal. While the flat-foot squat “naturally” approached the hypothesised optimal birth conditions, none of the participants in that study achieved the biomechanically “advantageous” position with both a flattened lumbar spine and a pelvic inlet plane perpendicular to the spinal axis, that is considered ideal for foetal descent [45,46,71]. Achieving such alignment would have required participants to adopt a more kyphotic posture, which was not observed spontaneously, suggesting a mismatch between biomechanical ideals and preferred or feasible movement strategies in pregnancy [45]. Hemmerich et al. [39] further reported that pregnant participants took longer to adopt the squatting position compared to non-pregnant controls, and exhibited greater hip flexion, abduction, and pelvic tilt, alongside lower peak hip extension moments, indicating altered load distribution and reduced mechanical efficiency.

Comparisons between squatting and supine positions (with and without manual lordosis correction) revealed no significant differences in measured lumbar curvature or hip kinematics, suggesting that compensatory strategies may not fully overcome pregnancy-related constraints [46]. In the McRoberts’ manoeuvre, varying the degree of thigh abduction prior to execution did not significantly affect lumbar lordosis or pelvic inclination [72].

As a whole, the findings suggest that achieving biomechanically “optimal” spinal and pelvic alignment in the tested birthing positions may be inherently constrained, even under controlled conditions [39,45,46,71]. The entire body chain, including thoracic spine configuration, foot positioning, and individual anatomical variability, must be taken into account [45,46]. Despite common assumptions, the McRoberts’ manoeuvre, regardless of initial thigh abduction, did not confer measurable biomechanical advantages in pelvic inclination or lumbar lordosis [72]. Similarly, when considering pelvic biomechanics alone, such as lumbar curvature and hip kinematics, squatting showed no significant superiority over supine positions, even with manual lordosis correction [46].

#### Gait and locomotor analysis.

**Spatiotemporal gait parameters:** Across studies, pregnancy is generally associated with reduced walking velocity, shorter step and stride lengths, increased step width, and longer double support times. These changes typically emerge from the second trimester and become more pronounced in the third [1,10,52,73–78]. They are generally interpreted as compensatory strategies to enhance stability in response to the anterior shift in the CoM and increasing physical demands [1,79].

Step width was found to increase progressively in several studies [1,55,79–81], with Lymbery and Gilleard [81] quantifying an average widening of 2.5 cm by late pregnancy. Increases in inter-ankle distance and base of support ratios were also reported, indicating lateral expansion to maintain medio-lateral stability [75].

However, findings are not entirely uniform. Some studies found no significant changes in walking velocity [50,80], step width [50,51,80,82], or stride length across gestation [75,83]. One study [84] even reported an increase in walking speed as pregnancy progressed, highlighting inter-individual variability and potential influences of habitual activity or context. Comparisons with non-pregnant controls showed reduced stride length in late pregnancy [56,78,85].

Cadence findings were similarly mixed: while some studies noted a decrease [10,80], others found it unchanged [75,77,83], suggesting individual differences in step frequency adjustments. Three longitudinal studies [43,75,86] confirmed that these spatiotemporal gait adaptations generally reverse postpartum, supporting the view that they are functional and transient rather than pathological. While the majority of studies have focused on walking, Bagwell et al. [87] extended this analysis to running biomechanics, reporting altered lower limb kinematics and joint loading patterns in pregnant and postpartum women compared to nulligravid controls. Their findings highlight that gait adaptations may extend beyond walking, affecting higher-impact locomotor tasks such as running.

**Pelvic and thoracic kinematics:** Pregnancy induces a range of modifications in pelvic and thoracic kinematics, with some adaptations emerging as early as the second trimester [43,75,88] and others varying with the presence of pelvic girdle pain (PGP) [86,89]. Pelvic tilt tends to increase anteriorly with gestational age, particularly during walking [1,43,55,75,80,88]. Forczek et al. [75] also reported increased pelvic range of motion (RoM) between trimesters, especially in the transverse plane. Conversely, women with PGP demonstrated reduced frontal and transverse plane pelvic RoM, decreased lateral pelvic translation, and diminished thoracic rotation, suggesting a compensatory or protective adaptation [86]. Similar patterns (reduced pelvic RoM in the frontal and transverse planes and diminished thoracic rotation) have also been observed in asymptomatic pregnant women compared to non-pregnant controls, suggesting that these adaptations are not solely pain-driven but part of the broader biomechanical adaptations during pregnancy [80] (Gilleard, 2013). At the hip joint, women with PGP showed reduced sagittal and frontal plane RoM, even after accounting for gait speed and stride length [86].

Trunk and thoracic segment co-ordination also changes: McCrory et al. [52] identified altered thoracic co-ordination patterns in pregnant fallers, possibly reflecting shifts in motor control strategies, while Wu et al. [89] found disrupted timing and reduced co-ordination between horizontal trunk and pelvic rotations in women with PGP, suggesting further neuromechanical compensations specific to pain-related gait alterations.

**Plantar pressure and foot loading:** Pregnancy alters plantar pressure distribution during gait, prompting adaptations that may influence foot stability, arch support, and balance. Karadag-Saygi et al. [77] and Vatansever et al. [90] have reported increased peak pressures in the forefoot and midfoot regions, correlating with foot pain and discomfort. Masłoń et al. [91] demonstrated progressive increases in medial forefoot and midfoot loading across trimesters, accompanied by flattening of the medial longitudinal arch. Mikeska et al. [92] further confirmed increased plantar pressures and reported trimester-related changes in arch index and foot posture, supporting a gradual structural adaptation of the foot. Alterations in pressure trajectories have also been observed. Lymbery and Gilleard [81] noted medial-lateral shifts in pressure trajectories, possibly linked to balance control. Zhang et al. [93] identified changes in centre of pressure (CoP) progression and a reduction in propulsion velocity, suggesting compromised push-off mechanics. These findings align with earlier observations of arch flattening during pregnancy [40,94], which may contribute to foot discomfort and altered gait mechanics. Footwear design tailored to redistribute plantar pressure, as investigated by Gimunová et al. [95], showed some benefits in gait symmetry and comfort. Ramachandra et al. [96] observed a progressive increase in plantar contact area in the forefoot and medial midfoot regions as gestation advanced, accompanied by a significant reduction in dynamic arch height, indicating progressive flattening of the foot. These findings are reinforced by Casto et al. [97], who found that plantar pressure distribution in the third trimester differed not only in magnitude but also in spatial location. Pregnant participants showed significantly higher peak pressures in the midfoot and forefoot during walking and altered CoP trajectories, indicating delayed push-off and reduced propulsive efficiency.

**Fall risk and neuromechanical adaptations:** A distinct subset of studies investigated gait in the context of fall risk by comparing pregnant fallers to non-fallers. McCrory et al. [52] reported that fallers exhibited significantly reduced gait speed and increased step width, while McCrory et al. [56] found altered thoracic segment co-ordination patterns, suggesting compromised trunk control. These maladaptive compensations may reflect attempts to stabilise gait under increased biomechanical demands [52,56]. Bagwell et al. [98] and Catena et al. [99] further linked such changes to diminished dynamic balance control and reduced CoP excursion. Flores et al. [100] added that walking balance and postural stability on a treadmill decline progressively throughout gestation, particularly in the third trimester, with increased CoM sway and variability in step placement, indicating a growing instability risk under constrained locomotor conditions. Zia et al. [78] and Abedzadehzavareh and Catena [82] highlighted the biomechanical characteristics of waddling gait, which may enhance mediolateral stability but reduce energy efficiency. Despite these adaptations, the biomechanical mechanisms underpinning falls in pregnancy remain multifactorial and incompletely understood.

**Neuromuscular control and joint-level adaptations:** Electromyographic and kinetic studies, including Music et al. [84], Bagwell et al. [98] and Bagwell et al. [101], reported greater hip and knee extensor muscle activity and altered joint kinetics during gait in late pregnancy. Specifically, reductions in ankle joint power and knee extensor moments, alongside increased hip extensor demand, suggest compensatory neuromuscular strategies to accommodate changes in posture, load distribution, and RoM. Bagwell et al. [98] also highlighted altered muscle activation patterns throughout pregnancy and into the postpartum period, underscoring continued neuromuscular adaptation beyond delivery. Catena et al. [99] also found that reduced hip extension and ankle plantarflexion RoM were associated with diminished balance control, reinforcing the need for coordinated joint-level responses to preserve dynamic stability. Branco et al. [44] further suggested that increased body mass and fat distribution contribute to elevated joint loading and altered kinetic profiles, particularly in the hip and ankle joints. Daneau et al. [102] added that trunk stiffness and damping characteristics change across pregnancy in response to hormonal and musculoskeletal factors, and these neuromechanical properties were significantly associated with clinical pain outcomes, highlighting the contribution of active trunk control mechanisms to maternal musculoskeletal health.

#### Functional tasks and interventions.

**Functional tasks:** Functional movements such as sit-to-stand transitions, stair negotiation, and load-carrying become biomechanically demanding during pregnancy due to increased body mass, anteriorly shifted CoM, and altered joint mobility and muscle function. Evidence suggests that pregnant women adopt compensatory strategies across multiple tasks to preserve stability and manage rising mechanical demands, particularly in late gestation.

Sit-to-stand and stand-to-sit transitions have been extensively studied as they require co-ordinated control of trunk and lower limb segments. Pregnancy-related adaptations reduce sagittal plane motion and redistribute joint loading. For example, Lou et al. [103] and Catena et al. [104] found reduced hip extension and trunk flexion angles in late pregnancy, alongside diminished hip joint moments, suggesting a shift in mechanical demand towards the knee and ankle. Sunaga et al. [60] and Sunaga et al. [105] demonstrated altered inertial parameters of the lower trunk and increased trunk flexion velocities during sit-to-stand tasks, highlighting the influence of changing body segment dynamics on transitional control. Gilleard et al. [49] similarly reported increased trunk flexion angles, longer task duration, and greater variability in lower limb kinematics during sit-to-stand, changes that persisted into postpartum. Takeda et al. [58] showed that using a handrail significantly reduced peak knee and hip moments and improved balance during sit-to-stand in the third trimester, suggesting that simple ergonomic aids may support movement efficiency. In a complementary analysis of the reverse movement, Takeda [57] observed altered muscle activation timing and joint co-ordination during stand-to-sit, indicating continued neuromechanical adaptations for controlled descent. Although this review focuses on the antenatal period, some evidence suggests that pregnancy-related adaptations may persist postpartum. Chu et al. [106] observed continued compensatory sit-to-stand patterns after delivery, while Eckland et al. [107] identified lingering inefficiencies in trunk co-ordination

Stair negotiation also presents challenges. Ascent and descent require precise control of limb trajectory and dynamic balance. McCrory et al. [53] and McCrory et al. [54] reported increased vertical ground reaction forces (GRFs) and longer double-support times during stair descent in pregnant women, suggesting a cautious motor strategy aimed at fall prevention. Complementing these findings, Takeda et al. [108] observed a shift from ankle-dominant to hip-dominant strategies when managing stairs, indicating altered neuromuscular co-ordination to preserve equilibrium.

Walking initiation and transitional movements, such as the first steps from a static position, demand anticipatory control of posture. Zia et al. [78] and Sunaga et al. [105] both documented increased variability in step placement and CoP progression, alongside delayed activation of stabilising muscles. These findings suggest a disruption in the timing and co-ordination of anticipatory mechanisms. Dumas et al. [62] further reported elevated muscular effort in the trunk and lower limbs during transitional tasks, reinforcing the view that movement initiation becomes more physically demanding as pregnancy progresses.

Load-carrying tasks, especially under dual-load conditions, add further complexity to postural control and musculoskeletal demands. Dumas et al. [62] developed a biomechanical model of the pregnant trunk and showed that carrying anterior or asymmetrical loads significantly increased spinal extensor and hip joint demands compared to non-pregnant conditions, with spinal moments amplified by both gestational posture and load configuration. These findings reinforce the role of compensatory muscle strategies and highlight how load carriage can exacerbate mechanical strain during pregnancy. Beaucage-Gauvreau et al. [61] examined head load carriage in pregnant women and found pronounced lumbar lordosis and thoracic extension, potentially reflecting compensatory adjustments to offset anterior CoM displacement. Gilleard et al. [47] also observed increased trunk muscle activity and altered static posture during seated tasks, implicating muscular fatigue and postural strain as possible contributors to low back pain (LBP) during pregnancy.

Lastly, task-specific balance responses highlight the impact of pelvic instability and musculoskeletal adaptation. De Groot et al. [109] and Christensen et al. [110] found impaired pelvic stability and altered motor control during tasks like the active straight leg raise (ASLR) in women with PGP. Bey et al. [111] reported structural changes in the vastus lateralis that may influence functional neuromuscular performance. Bagwell et al. [112] described reduced sway, slower sway velocity, and lower sample entropy in both medio-lateral and anteroposterior directions during single-leg stance across pregnancy, particularly in the second and third trimesters, suggesting a more rigid and repetitive postural control strategy. Shorter stance time and higher frequency responses in late pregnancy and even postpartum further indicated limited adaptability.

**Interventions:** Only three studies directly evaluated interventions to support functional movement during pregnancy, yet they provide early insights into how targeted aids or exercise modifications may alleviate biomechanical strain and enhance safety. Bayraktar et al. [113] found that using a handrail during sit-to-stand in the third trimester significantly reduced lower limb joint moments, improved balance, and shortened transition time, suggesting that simple supports can ease mechanical demands and enhance movement efficiency. Zurawski et al. [114] tested an antenatal exercise programme and observed improved trunk stability and reduced CoP sway during dynamic tasks, highlighting the value of targeted physical activity in preserving motor function and minimising compensatory patterns. In a water-based exercise study, Alberton et al. [115] showed that aquatic immersion significantly reduced GRFs during walking and marching activities, suggesting that such environments may offer a safer, low-impact option for physical activity during pregnancy by decreasing mechanical load on the musculoskeletal system.

**RoM-specific tasks:** Three studies have examined RoM-specific tasks to investigate segmental mobility and neuromuscular adaptations during pregnancy, particularly in the lumbar spine and pelvis. De Groot et al. [109] used the ASLR to assess load transfer across the pelvis, finding that women with PGP showed greater pelvic rotation and impaired force closure, indicating compromised stability. Gilleard et al. [47] examined seated posture and reported increased anterior pelvic tilt and reduced lumbar lordosis during sitting in pregnancy, suggesting that static postural control is also affected by gestational changes. Bivia-Roig et al. [42] assessed lumbar motion patterns and found altered amplitude and co-ordination of trunk flexion and extension, along with modified erector spinae muscle activity, especially in the third trimester, with some changes persisting postpartum. These studies highlight that RoM-specific tasks can sensitively detect lumbopelvic adaptations and may help identify individuals at risk of reduced function or musculoskeletal discomfort.

#### Balance and stability.

Maintaining balance during pregnancy becomes increasingly challenging due to increased body mass, anterior CoM shift, and neuromuscular adaptations affecting postural control. Across gestation, both static and dynamic balance are compromised, particularly in the third trimester. Opala-Berdzik et al. [9] and Oliveira et al. [116] reported significant increases in CoP displacement and reduced static stability, with only partial recovery postpartum. McCrory et al. [117] observed similar trends in dynamic conditions, including longer recovery times and larger CoP excursions, suggesting impaired neuromuscular responsiveness and a shift from ankle-dominant to hip-dominant control. Jang et al. [118] noted that pregnant women tend to overestimate their balance ability, which may increase fall risk. To enhance stability, women often adopt a wider stance, yet this “waddling” strategy may be insufficient in complex or unstable environments [55,82].

Studies of neuromuscular activity support these findings. Moreira et al. [119] observed increased myoelectric activity in postural muscles such as the erector spinae and gastrocnemius during quiet standing in the third trimester, suggesting heightened muscular effort is required to maintain postural equilibrium. Bivia-Roig et al. [41] similarly documented altered trunk posture and muscle activity in pregnancy and postpartum, reinforcing that motor strategies for upright stance continue to adapt beyond delivery. Bagwell et al. [120] further showed that postural control characteristics in early pregnancy, specifically altered CoP parameters and reduced stability, were predictive of developing LBP or PGP later in gestation or postpartum. These findings highlight the potential clinical value of early balance assessment for identifying women at greater risk of musculoskeletal complications. Further biomechanical insights were provided by Catena et al. [59], who reported significant shifts in whole-body CoM location and altered postural alignment, particularly in the sagittal plane. These kinematic changes, in combination with foot pressure redistribution, contribute to discomfort and instability. Karadag-Saygi et al. [77] quantified elevated forefoot plantar pressure and self-reported foot pain in the third trimester, a pattern that not only affects stability but may also disrupt normal stance patterns and walking.

### Biomechanics of labour

Despite targeted searches and eligibility criteria allowing for studies conducted during labour, no studies were identified that investigated biomechanical outcomes involving labouring women. Accordingly, this section is not developed further as no evidence was available to synthesise.

## Discussion

Given the absence of prior synthesis, this scoping review was essential to establish the scope of existing biomechanical research in pregnancy and labour and to identify key knowledge gaps for future investigation. It provides the most comprehensive synthesis to date, mapping 87 peer-reviewed studies and identifying key domains on several antenatal adaptations, changes, and responses across posture, gait, functional tasks, and balance. Although it offers important knowledge, it also reveals substantial, critical, and urgent gaps in our understanding.

### Antenatal biomechanics

Across all domains, the evidence consistently demonstrated that pregnancy elicits wide-ranging biomechanical adaptations across posture, gait, functional movements, and balance. However, a key theme that emerged is the absence of a one-size-fits-all trajectory. Most adaptations appear to be highly individualised, influenced by gestational stage, body morphology, presence of pain (e.g., PGP), task type, and environmental context.

Although often assumed to increase uniformly, lumbar lordosis shows no consistent pattern across gestation, with some individuals exhibiting increased curvature [63], others showing a decrease [64], or no change [41,48,64], with even intra-sample variability reported [38]. Similarly, pelvic orientation varied between studies [65,66], highlighting the diversity of compensatory strategies rather than a uniform biomechanical shift. Common gait changes during pregnancy include increased step width and reduced walking velocity and stride length (e.g., [1,10,75,76]), though findings are inconsistent, with some studies reporting no changes (e.g., [51,75,80,83]), and some individuals displaying unexpected patterns such as increased walking speed in late pregnancy [84]. Similar inconsistencies are seen for parameters like cadence [10,75,77,80,83] and pelvic RoM [75,86]. These inconsistencies suggest that gait adaptations are not universal but reflect diverse coping strategies. Similarly, functional movements such as sit-to-stand transitions, stair negotiation, and load carriage are performed differently during pregnancy, with individuals redistributing joint loading and relying more on neuromuscular control [103,112]. These adaptations often become more pronounced in late gestation and may persist postpartum [80,107], but their extent and clinical relevance vary considerably between individuals. This variability likely reflects differing strategies to accommodate the anterior shift in the CoM, shaped not only by individual factors but also by discomfort, fatigue, and underlying musculoskeletal conditions [52,56,84,101], or metabolic conditions [69].

### Critical appraisal of the evidence and methodological limitations

This review identified that biomechanical research in pregnancy began to grow notably only after 2008. Several factors may explain this delay. Although biomechanics has its roots in Ancient Greece, with Aristotle (384–322 B.C.) describing the body as a mechanical system in his work *De Motu Animalium* (On the Movement of Animals), modern biomechanical investigations were limited for centuries. In the 1800s and 1900s, early pioneers explored human musculoskeletal biomechanics using photography and rudimentary motion analysis techniques [121]. However, the lack of advanced technology constrained the field’s development. It was not until the 1990s that modern motion capture systems began to be introduced into research and clinical practice, enabling more precise and systematic study of human movement [122,123]. Despite this, biomechanical knowledge in other fields is vast, has grown significantly, and continues to expand [124–126]. However, as shown by this review, it remains limited in the context of pregnancy. One contributing factor may be that the inclusion of women in research and clinical trials only became a law in 1993 [127,128]. Furthermore, it has been repeatedly reported that women’s health research remains chronically underfunded, receiving a disproportionately small share of public research funding, sometimes less than 2.5% [129–133].

For the majority of studies, a notable concern is the widespread reliance on small, convenience-based samples, often without power calculations or justification for sample size. Such limitations restrict statistical power and reduce the ability to detect subtle but potentially meaningful biomechanical differences. Additionally, several studies appeared to be based on overlapping participant cohorts, which may exaggerate the perceived breadth and diversity of the evidence base (e.g., [47–49,52–54]). On a positive note, over 40% of the studies used longitudinal designs with at least two or three antenatal follow-ups, and in some cases, an additional postnatal assessment (e.g., [44,48,60,118]). This is a valuable strength, as it allows for the examination of temporal adaptations across the antenatal period, with pregnancy being a dynamic, time-dependent process.

Reporting of participant demographics was inconsistent. While most studies reported height, weight and/or BMI, and gestational age, most omitted key variables such as parity, physical activity levels, or comorbidities. The most critical missing variable was ethnicity. Only one study explicitly reported the ethnic background of its participants [59], and a few others implied regional origin without providing meaningful demographic or ethnic detail [60–62]. As a result, no study included a racially or ethnically diverse sample, and none examined whether biomechanical parameters varied by ethnicity, socioeconomic status, or cultural context. This omission not only limits the generalisability of findings but raises important concerns about equity and the representation of ethnically diverse pregnant populations in biomechanics research. This is particularly problematic given documented anatomical and physiological differences across population groups that may influence pregnancy biomechanics, such as variations in pelvic morphology [23,134,135] and joint mobility [136,137]. For instance, sacral slope has been reported to be approximately 3.6° larger in Caucasian women compared to Asian women [134,135]. East Asian women have also been observed to have reduced pelvic organ mobility in the anterior and posterior vaginal compartments [138] and Asian populations show the largest degree of age-related change in spinopelvic parameters compared to other groups, likely due to lower pelvic incidence [139]. The lack of knowledge about ethnic diversity in perinatal care from a biomechanical perspective, combined with recent findings that known risk factors do not fully explain ethnic disparities in maternal mortality [140], may partly explain and contribute to why mortality rates [140,141] and adverse pregnancy or birth outcomes [142] remain disproportionately high for Black and Asian women compared to White women. For instance, MBRRACE-UK reports show that Black women in the UK face nearly three times the risk of maternal death compared to White women [143,144]. This makes it extremely urgent to address these gaps and better understand ethnic variations related to antenatal biomechanics.

Geographically, studies were disproportionately concentrated in HICs, particularly the USA and parts of Europe. Only a small number originated from LMICs, despite the fact that these areas often experience higher rates of maternal morbidity and mortality [26,145]. This lack of global representation, combined with the near-total absence of knowledge about ethnic diversity in pregnancy biomechanics, limits the applicability, generalisability, and equity of current findings in informing universal antenatal care.

Methodologically, the dominance of kinematic and kinetic assessments reflects the maturity of the biomechanical field, yet notable limitations remain. While motion capture systems, force plates, and pressure mapping were widely used, sEMG and real-time neuromuscular assessments were underutilised. Few studies employed integrated, multi-modal approaches, such as combining motion analysis with neuromuscular or physiological measurements (e.g., [41,109,119]). Moreover, very few linked biomechanical findings to clinical symptoms or outcomes (e.g., pain, fall risk, or birth outcomes) (e.g., [56,86,98,110]), reducing their translational value. Without these connections, it remains unclear how biomechanical adaptations relate to maternal well-being or clinical decision-making. There is an urgent need for future research to move beyond describing *what* changes occur and *how*, to also asking *so what*; by explicitly linking biomechanical findings to clinical, functional, or obstetric outcomes. This highlights the importance of interdisciplinary collaboration that connects biomechanical data with lived experience and healthcare delivery.

Additionally, the overwhelming focus on controlled laboratory environments, often using static, constrained protocols, raises questions about ecological validity. Few studies considered real-world functional demands or environmental variability, limiting our understanding of how pregnant women adapt in everyday contexts. Finally, the most significant gap identified in this review is the complete absence of studies investigating the biomechanics of labour itself; a critical omission that warrants dedicated attention in the following section.

### The missing science of labour

This review identified a striking and concerning gap: not a single study to date has investigated the biomechanics of labour. Despite global concern over maternal morbidity, mortality, and birth-related complications [26,145,146], no empirical research has captured biomechanical data from labouring women. This absence limits our understanding of how posture, movement, clinical manoeuvres, and mechanical forces interact during labour, hindering the development of evidence-informed guidance for maternal positioning, physical support, and risk management during childbirth.

Labour represents one of the most biomechanically complex events in the human lifespan. It involves coordinated neuromuscular effort, dynamic pelvic motion, maternal–foetal interactions, and significant mechanical loading across the musculoskeletal system [18,147,148]. Yet, in contrast to other fields [124–126], labour remains essentially unmeasured from a biomechanical perspective. We are, in effect, operating in a biomechanical evidence vacuum when supporting birth, as no empirical studies to date have investigated or described the dynamic biomechanical mechanisms of labour.

The only available insights come from five small experimental studies that assessed birthing positions in non-labouring, late-pregnant individuals under static, controlled conditions [39,45,46,71,72]. These studies could not account for the real-time mechanical interplay of uterine contractions, maternal pushing, or foetal descent. Still, they provide a critical reality check for widely held clinical assumptions. Despite the common belief that positions like squatting or the McRoberts’ manoeuvre optimise pelvic mechanics [20,72,149,150], this limited body of antenatal evidence offers no biomechanical support for the superiority of any investigated position [39,45,46,71,72]. Squatting, for instance, demonstrated substantial interindividual variability and failed to produce the theorised “optimal” pelvic configuration in any participant studied. Even with manual lordosis correction or altered foot placement, no one achieved the alignment described in clinical guidance [45,46,71]. Notably, comparisons between squatting and supine positions, both with and without manual lordosis correction, revealed no significant differences in lumbar curvature or hip kinematics, suggesting that pregnancy-related biomechanical constraints may not be easily overcome by positional adjustments alone [46]. Similarly, the McRoberts’ manoeuvre, despite its routine use in obstetric emergencies, did not significantly affect pelvic inclination or lumbar lordosis, regardless of thigh abduction [72].

While these studies are limited by sample size and were conducted outside of labour, their findings challenge the biomechanical assumptions underpinning routine obstetric care. If no measurable advantages are observed even in controlled antenatal conditions, it is plausible that the perceived benefits of certain positions stem more from individual comfort, maternal agency, or midwifery facilitation than from mechanical optimisation [151–153]. All commonly used manoeuvres, manual handling techniques, and positional assumptions remain biomechanically unverified. These practices are based on tradition and clinical observation [150,154,155], not on direct biomechanical evidence. Until we study childbirth biomechanics directly, during labour, capturing maternal–foetal dynamics, contractions, and maternal effort, we must exercise caution in asserting mechanical “truths” about labour [21]. Clinical humility and personalised, responsive care remain the strongest tools. This underscores the urgent need for biomechanical research conducted during actual labour, capturing the dynamic influence of contractions, foetal descent, and maternal effort, to meaningfully inform evidence-based positioning guidance. It also reinforces that maternal positioning should remain individualised rather than protocolised, informed by broader functional and physiological considerations beyond lumbopelvic alignment alone, respecting the complexity and variability of maternal anatomy and movement, and prioritising safety and well-being.

This persistent void in labour biomechanics is likely due, in part, to the methodological and ethical complexities of conducting research during childbirth. From a technical standpoint, conventional tools used in biomechanical analysis, particularly those for capturing kinematic and kinetic data, pose significant challenges in intrapartum settings. Kinematic analysis typically requires the placement of multiple reflective markers on the body, which must remain visible and unobstructed. This is often impractical during labour, especially in certain positions where skin access is limited and movement is dynamic, assisted, and frequently involves close contact with healthcare staff. These systems also require considerable spatial setup, including approximately a dozen synchronised cameras, tripods, and processing units; equipment that is impractical to install in most clinical labour environments without disrupting care [21,45,72,156,157]. Even markerless motion capture systems, while less invasive, currently offer limited accuracy and still require similar infrastructure [158].

While computational modelling has offered some biomechanical insight, its scope remains constrained by simplifications, assumptions, and a lack of empirical validation. Most models are subject-specific, often representing only isolated components such as the pelvis, and fail to capture the integrated, chain-like nature of human movement. Furthermore, key inputs, such as soft tissue properties, uterine contractile force, or foetal interaction, are either assumed or not computable, limiting the ecological validity and generalisability of findings [19,20,159–161]. Consequently, experts in the field continue to emphasise the need for *in vivo* data, or at a minimum, studies that integrate both *in vivo* and *in silico* approaches to improve biomechanical realism and relevance [19,20].

However, the complete absence of even preliminary or feasibility studies in real-world labour settings points to more than technical difficulty. It suggests a deeper structural neglect of this research area. The implications of this omission are significant. Without direct biomechanical data from labouring individuals, the development of evidence-based guidance on positioning, manual support, or intervention strategies remains speculative from a mechanical perspective. Moreover, the ability to understand how individual variations in anatomy, posture, or movement during labour affect clinical outcomes, such as labour duration, mode of birth, perineal trauma, or neonatal compromise, is severely limited [162–166]. Given that the biomechanics of labour directly impact both maternal and neonatal safety, this represents a missed opportunity to inform and improve perinatal outcomes [15,20]. Urgent research investment is needed to develop safe, ethical, and feasible methodologies that can capture the mechanical realities of childbirth *in situ*. Without this, the biomechanical dimension of labour will remain invisible, and the assumptions currently guiding clinical care will remain largely untested.

### Implications for research and practice

While advances have been made in identifying the physiological mechanisms of pregnancy and childbirth, biomechanical insights have not yet been fully leveraged to improve clinical outcomes, inform practice, or shape policy. This gap is particularly critical given the complex interplay between mechanical forces, maternal anatomy, labour progression, and the failure to improve mortality and morbidity rates.

For researchers, this review underscores the need to move beyond descriptive studies and towards more integrative, hypothesis-driven investigations that link biomechanical patterns to clinically relevant outcomes to improve the translational value of findings, support perinatal practice and wellbeing, and help identify women at risk of musculoskeletal complications. There is an urgent need to develop methodologies and means that allow real-world data acquisition, especially during labour. Participant diversity, individual variability, particularly in relation to ethnicity, and differences between ethnicities should be accounted for in any future studies. Understanding the mechanical realities of labour could inform decisions about birthing positions, assistive tools, or manual techniques used in clinical practice. As maternal morbidity and mortality remain unacceptably high globally, biomechanical research must become part of the broader agenda for improving perinatal outcomes.

From a clinical perspective, the findings underscore the need for clinicians to develop a working understanding of pregnancy-related biomechanical changes. These insights can support the interpretation of physical symptoms, guide safe mobility and posture strategies, and inform timely referrals to specialist care, such as physiotherapy. Biomechanical adaptations during pregnancy are clearly multi-faceted, involving complex interactions between posture, movement, stability, and neuromuscular control. These adaptations are not uniform and appear to be highly individualised, influenced by factors such as gestational stage, body morphology, symptom presence, and task demands. Recognising this heterogeneity is crucial for informing personalised antenatal care and developing targeted interventions to support function and reduce physical strain. However, the complete lack of *in vivo* biomechanical evidence for intrapartum positions or manual techniques highlights a critical missed opportunity to support safer, more efficient, and more personalised birth. It also signals caution: common practices, including positioning, manual support, and manoeuvres, remain largely unverified from a mechanical standpoint. Without direct evidence, clinicians must avoid over-relying on tradition or theoretical assumptions. Instead, care should remain individualised, prioritising maternal comfort, agency, and safety, while maintaining clinical humility in the absence of empirical biomechanical truths. Bridging the gap between biomechanical insight and clinical application is essential for improving outcomes and empowering women throughout the perinatal journey.

### Limitations of the review

Although the search strategy was comprehensive and covered multiple databases, only studies published in English were eligible for inclusion. This may have resulted in the omission of relevant research published in other languages. As a scoping review, we did not conduct a formal quality appraisal or risk of bias assessment of the included studies. While this approach is consistent with scoping review methodology, it limits our ability to comment on the strength or reliability of individual study findings beyond descriptive and narrative synthesis. Finally, data extraction was constrained by the completeness and clarity of reporting in the original publications. Inconsistent reporting of gestational age, anthropometric characteristics, and methodological details posed challenges for cross-study comparison and synthesis.

## Conclusion

This scoping review mapped the current landscape of biomechanical research in pregnancy and labour. It identified a wide range of antenatal adaptations across posture, gait, functional tasks, and balance, reflecting the complex and individualised nature of physical change during pregnancy. However, the review also exposed substantial gaps, including limited methodological diversity, minimal integration with clinical and health outcomes, under-representation of ethnically and socioeconomically diverse populations, and the complete absence of studies investigating biomechanical processes during labour. Together, these findings highlight the urgent need for more inclusive, longitudinal, and outcome-oriented research that not only captures what biomechanical adaptations occur, but also how they relate to maternal well-being, clinical care, and antenatal outcomes. In particular, addressing the missing science of labour biomechanics and improving ethnic representation are critical next steps in advancing antenatal health equity and safety. As maternal morbidity and mortality remain pressing global issues, the integration of biomechanical insight into clinical and research practice is not only timely but essential.

## Supporting information

S1 Table Study characteristics and charting of extracted data from the 87 included records. The full dataset charted during the review, including author(s), year, country, study design, participant characteristics, methods, outcomes, and key findings.
(XLSX)

S1 Appendix Search strategy. Full database search strategy, including search terms, syntax, and number of records retrieved in MEDLINE, EMBASE, and MIDIRS.
(DOCX)

S2 Appendix Records unavailable in full text. List of records unavailable in full text and therefore excluded from the review.
(DOCX)

S3 Appendix PRISMA-ScR Checklist. Completed PRISMA-ScR checklist detailing adherence to reporting standards for scoping reviews.
(DOCX)
