# Supplementary material for: What do we actually know about the biomechanics of pregnancy and labour? A systematic scoping review
Source: PLoS One. 2025 Dec 1;20(12):e0337595. doi: 10.1371/journal.pone.0337595 (PMC12668629; doi:10.1371/journal.pone.0337595)
Supplement: S1 Appendix — Full database search strategy, including search terms, syntax, and number of records retrieved in MEDLINE, EMBASE, and MIDIRS. (DOCX) [file pone.0337595.s002.docx]

**Appendix 1 – Search Strategy and Search Terms

Medline**

Database: Ovid MEDLINE(R) ALL <1946 to May 01, 2025>

Date searched: 2025-05-02

1. Biomechanic*.mp. [mp=title, book title, abstract, original title, name of substance word, subject heading word, floating sub-heading word, keyword heading word, organism supplementary concept word, protocol supplementary concept word, rare disease supplementary concept word, unique identifier, synonyms, population supplementary concept word, anatomy supplementary concept word]

Hits: 194038

2. Motion capture.mp. [mp=title, book title, abstract, original title, name of substance word, subject heading word, floating sub-heading word, keyword heading word, organism supplementary concept word, protocol supplementary concept word, rare disease supplementary concept word, unique identifier, synonyms, population supplementary concept word, anatomy supplementary concept word]

Hits: 7463

3. Motion analysis.mp. [mp=title, book title, abstract, original title, name of substance word, subject heading word, floating sub-heading word, keyword heading word, organism supplementary concept word, protocol supplementary concept word, rare disease supplementary concept word, unique identifier, synonyms, population supplementary concept word, anatomy supplementary concept word]

Hits: 8801

4. Movement analysis.mp. [mp=title, book title, abstract, original title, name of substance word, subject heading word, floating sub-heading word, keyword heading word, organism supplementary concept word, protocol supplementary concept word, rare disease supplementary concept word, unique identifier, synonyms, population supplementary concept word, anatomy supplementary concept word]

Hits: 1808

5. Body tracking.mp. [mp=title, book title, abstract, original title, name of substance word, subject heading word, floating sub-heading word, keyword heading word, organism supplementary concept word, protocol supplementary concept word, rare disease supplementary concept word, unique identifier, synonyms, population supplementary concept word, anatomy supplementary concept word]

Hits: 118

6. or/1-5

Hits: 203229

7. Childbirth.mp. [mp=title, book title, abstract, original title, name of substance word, subject heading word, floating sub-heading word, keyword heading word, organism supplementary concept word, protocol supplementary concept word, rare disease supplementary concept word, unique identifier, synonyms, population supplementary concept word, anatomy supplementary concept word]

Hits: 30771

8. Birth.mp. [mp=title, book title, abstract, original title, name of substance word, subject heading word, floating sub-heading word, keyword heading word, organism supplementary concept word, protocol supplementary concept word, rare disease supplementary concept word, unique identifier, synonyms, population supplementary concept word, anatomy supplementary concept word]

Hits: 432382

9. Labo?r.mp. [mp=title, book title, abstract, original title, name of substance word, subject heading word, floating sub-heading word, keyword heading word, organism supplementary concept word, protocol supplementary concept word, rare disease supplementary concept word, unique identifier, synonyms, population supplementary concept word, anatomy supplementary concept word]

Hits: 179209

10. Pregnan*.mp. [mp=title, book title, abstract, original title, name of substance word, subject heading word, floating sub-heading word, keyword heading word, organism supplementary concept word, protocol supplementary concept word, rare disease supplementary concept word, unique identifier, synonyms, population supplementary concept word, anatomy supplementary concept word]

Hits: 1206068

11. or/7-10

Hits: 1510980

12. 6 and 11

Hits: 1940

13. exp animals/ not humans.sh.

Hits: 5340119

14. 12 not 13

Hits: 1510

15. limit 14 to english language

Hits: 1402

**Embase**

Database: Embase (Ovid) <1974 to 2025 May 01>

Date searched: 2025-05-02

1. Biomechanic*.mp. [mp=title, abstract, heading word, drug trade name, original title, device manufacturer, drug manufacturer, device trade name, keyword heading word, floating subheading word, candidate term word]

Hits: 198111

2. Motion capture.mp. [mp=title, abstract, heading word, drug trade name, original title, device manufacturer, drug manufacturer, device trade name, keyword heading word, floating subheading word, candidate term word]

Hits: 9174

3. Motion analysis.mp. [mp=title, abstract, heading word, drug trade name, original title, device manufacturer, drug manufacturer, device trade name, keyword heading word, floating subheading word, candidate term word]

Hits: 21037

4. Movement analysis.mp. [mp=title, abstract, heading word, drug trade name, original title, device manufacturer, drug manufacturer, device trade name, keyword heading word, floating subheading word, candidate term word]

Hits: 2494

5. Body tracking.mp. [mp=title, abstract, heading word, drug trade name, original title, device manufacturer, drug manufacturer, device trade name, keyword heading word, floating subheading word, candidate term word]

Hits: 136

6. or/1-5

Hits: 218394

7. Childbirth.mp. [mp=title, abstract, heading word, drug trade name, original title, device manufacturer, drug manufacturer, device trade name, keyword heading word, floating subheading word, candidate term word]

Hits: 50168

8. Birth.mp. [mp=title, abstract, heading word, drug trade name, original title, device manufacturer, drug manufacturer, device trade name, keyword heading word, floating subheading word, candidate term word]

Hits: 593072

9. Labo?r.mp. [mp=title, abstract, heading word, drug trade name, original title, device manufacturer, drug manufacturer, device trade name, keyword heading word, floating subheading word, candidate term word]

Hits: 233119

10. Pregnan*.mp. [mp=title, abstract, heading word, drug trade name, original title, device manufacturer, drug manufacturer, device trade name, keyword heading word, floating subheading word, candidate term word]

Hits: 1187091

11. or/7-10

Hits: 1641102

12. 6 and 11

Hits: 2516

13. (rat or rats or mouse or mice or swine or porcine or murine or sheep or lambs or pigs or piglets or rabbit or rabbits or cat or cats or dog or dogs or cattle or bovine or monkey or monkeys or trout or marmoset$1).ti. and animal experiment/

Hits: 1298397

14. animal experiment/ not (human experiment/ or human/)

Hits: 2737302

15. 13 or 14

Hits: 2818523

16. 12 not 15

Hits: 2268

17. limit 16 to english language

Hits: 2130

**MIDIRS**

Database: Maternity & Infant Care Database (MIDIRS) (Ovid) <1971 to April 29, 2025>

Date searched: 2025-05-02

1. Biomechanic*.mp. [mp=abstract, subject heading word, title]

Hits: 130

2. Motion capture.mp. [mp=abstract, subject heading word, title]

Hits: 10

3. Motion analysis.mp. [mp=abstract, subject heading word, title]

Hits: 5

4. Movement analysis.mp. [mp=abstract, subject heading word, title]

Hits: 5

5. Body tracking.mp. [mp=abstract, subject heading word, title]

Hits: 0

6. or/1-5

Hits: 146

7. Childbirth.mp. [mp=abstract, subject heading word, title]

Hits: 18461

8. Birth.mp. [mp=abstract, subject heading word, title]

Hits: 95350

9. Labo?r.mp. [mp=abstract, subject heading word, title]

Hits: 42305

10. Pregnan*.mp. [mp=abstract, subject heading word, title]

Hits: 148542

11. or/7-10

Hits: 214421

12. 6 and 11

Hits: 106
