# Supplementary material for: What do we actually know about the biomechanics of pregnancy and labour? A systematic scoping review
Source: PLoS One. 2025 Dec 1;20(12):e0337595. doi: 10.1371/journal.pone.0337595 (PMC12668629; doi:10.1371/journal.pone.0337595)
Supplement: S2 Appendix — List of records unavailable in full text and therefore excluded from the review. (DOCX) [file pone.0337595.s003.docx]

|  | **Appendix 2: Records Unavailable in Full Text or Unretrievable by Authors** | | |
| --- | --- | --- | --- |
|  | **Author** | **Year** | **Title** |
| 1 | Botelho, S., Marques, J., Pereira, L., Lanza, A. H., Herrmann, V., Palma, P. and Riccetto, C. | 2011 | Can the abdominal and pelvic muscle training trigger the co-activation between the transversus abdominis / internal oblique muscle and the muscles of the pelvic floor during pregnancy and postpartum? |
| 2 | Aguiar, L., Vieira, F., Branco, M., Santos-Rocha, R. and Veloso, A. | 2012 | The overload influence in angular kinematics and joint moments in lower limb and pelvis during gait in sagittal plane |
| 3 | Desseauve, D., Fradet, L. and Pierre, F. | 2019 | What's news about thigh's abduction before McRoberts manoeuvre? An innovative biomechanical study |
| 4 | Foti, T., Davids, J. R. and Bagley, A. | 2000 | A biomechanical analysis of gait during pregnancy |
| 5 | Nyska, M., Sofer, D., Porat, A., Howard, C. B., Levi, A. and Meizner, I. | 1997 | Planter foot pressures in pregnant women |
| 6 | Sunaga, Y., Anan, M., Takahashi, M. and Shinkoda, K. | 2015 | Body segment inertial parameters estimation of the lower trunk of pregnant japanese women aimed at practical using in motion analysis |
| 7 | Zanetti, M. R. D., Resende, A. P. M., Petricelli, C. D., Nakamura, M. U., Pereira, S., Pereira, L. C. and Riccetto, C. L. Z. | 2011 | Electromyography activity of pelvic floor muscles: Is there any difference between nuliparous and primigravidae? |
| 8 | Pauk, J., Swinarska, D. and Daunoraviciene, K. | 2020 | Mechanisms of Gait Adaptation in Overweight Pregnant Women |
| 9 | Xu, S. | 2019 | Longitudinal evaluation of walking performance based on three-dimensional spine image during pregnancy and post-partum |
| 10 | Branco, M., Santos-Rocha, R., Aguiar, L., Vieira, F. and Veloso, A. | 2012 | Kinetic and kinematic analysis of gait of 2nd and 3rd trimester of pregnancy |
| 11 | Walker, C., Rodriguez, T., Herranz, A., Garcia, I. M., Espinosa, J. A., Sanchez, E. and Espuna, M. | 2011 | Second stage of labor with postural change and lateral position in women with epidural analgesia: A randomized controlled trial |
| 12 | Sokmez, F., Cetin, H., Bek, N., Kose, N. | 2018 | Relationship between vertebral curves, body mass index and foot arch height in pregnancy |
